# Ray Resection as a Personalized Surgical Technique for Progressive Hand Macrodactyly in a 60-Year-Old Patient: A Case Report and Literature Review

**DOI:** 10.7759/cureus.23357

**Published:** 2022-03-21

**Authors:** Argyris C Hadjimichael, Angelos Kaspiris, Sarantis Spyridonos

**Affiliations:** 1 Medical School, National and Kapodistrian University of Athens School of Medicine, Athens, GRC; 2 Upper Limb, Hand, and Microsurgery Department, "KAT" General Hospital, Athens, GRC; 3 Division of Orthopaedic Research, Laboratory of Molecular Pharmacology, School of Health Sciences, University of Patras, Patras, GRC

**Keywords:** median nerve, reconstruction, hypertrophy, hand, macrodactyly

## Abstract

Hand macrodactyly is a very scarce deformity. It was first described over 200 years ago and was characterized as “local gigantism” of one or multiple digits. Benign bone overgrowth, massive increase of soft tissue volume, and nerve involvement are associated with hand macrodactyly have been consistently reported in the literature. Often, macrodactyly affects one or more digits and is further classified as static or progressive, depending on the growth pattern, and as sporadic or syndromic, according to its genetic predisposition. Surgical treatment for hand macrodactyly remains a complex issue even for expert hand surgeons. In most of the cases, macrodactyly is diagnosed during early childhood and can be appropriately managed with minimal and well affordable surgical approaches that stabilize its fast progression. However, adults with progressive hand macrodactyly develop advanced deformities leading to severe functional deterioration and aesthetic hand dysmorphia. The purpose of this report is to document the management and surgical approach of the oldest published case, a 60-year-old adult patient with neglected progressive hand macrodactyly despite previous surgical attempts for disease stabilization. A personalized preoperative planning was created, which included ray resection involving the fourth metacarpal and fourthfinger along with extensive debulking of the overgrown fatty soft tissue and carpal tunnel release. At six months’ follow-up, the patient reported an excellent aesthetic and functional outcome.

## Introduction

The term “macrodactyly” is a descriptive term derived from the Greek words “macro” meaning long and “dactyl” meaning finger. Macrodactyly of the hand represents a very rare congenital deformity of unknown etiology, which constitutes less than 1% of congenital disorders in the upper extremity [1]. To our knowledge, hand macrodactyly affects approximately 1 out of 100,000 live births and can appear either as a sporadic (isolated form) or as part of a hereditary deformity syndrome (syndromic form) [2]. There are two distinct types of macrodactyly depending on the functional status of the hand. Static macrodactyly is the first type of macrodactyly, with affected fingers being roughly one and a half times the size compared to a normal finger [3]. Besides, static macrodactyly is present at birth and abnormal fingers grow in line with normal fingers [3]. Progressive macrodactyly constitutes the second type, causing continuous bony overgrowth even after skeletal maturity, with digits growing at a much faster rate compared to normal ones [3]. Oftentimes, the dysmorphic appearance of hand macrodactyly causes functional disability in the majority of cases along with cultural stigma, which might have a negative psychological impact on the patient [4].

A plethora of surgical interventions have been described mainly in young patients to cure static and prevent progressive hand macrodactyly, such as debulking procedures, epiphysiodesis, and osteotomies [1]. The purpose of this report is to document the personalized strategy for surgical reconstruction of a neglected progressive hand macrodactyly in an elder 60-year-old male patient - the oldest individual that we are aware of to have been surgically treated based on our literature search - and our efforts to obtain a functional hand with a good aesthetic outcome.

## Case presentation

**Figure 1:**
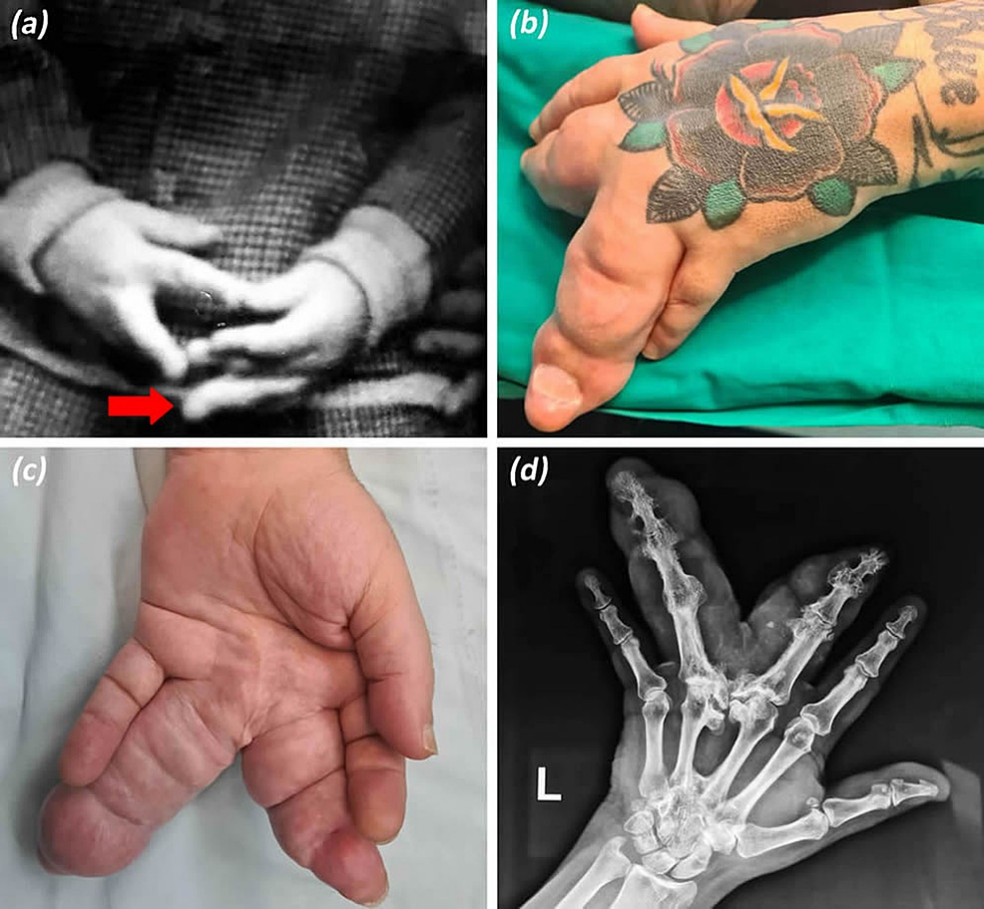
Preoperative images of the patient’s progressive hand macrodactyly. (a) Hand macrodactyly appeared during the patient’s early childhood (8 years) involving his left ring finger (arrow). (b and c) Dorsal and palmar hand views illustrate massive bone and soft tissue overgrowth of third and fourth macrodactyly digits. (d) Preoperative X-ray reveals enlarged phalanges as well as malalignment of osteoarthritic joints of third and fourth macrodactyly digits.

A 60-year-old male individual was presented to our department to seek consultation for his hand macrodactyly. The patient’s condition was diagnosed in early childhood as a sporadic isolated anomaly affecting moderately the middle and severely the ring fingers. According to his medical records, he underwent two minimal soft tissue debulking surgeries on the third interdigital space when he was 4 and 37 years old, respectively. Initially, he reported that his hypertrophic left middle and ring fingers became more painful and less functional recently, albeit he could manage it until then. The thumb, index, and little fingers were normal. Despite his hand deformity, the patient has been a professional guitar player for at least 40 years. During the last three years, he was complaining of progressive disproportionate growth of his middle and ring fingers (Figure 1). He was further experiencing numbness, tingling, and ache at the tip of all his left digits accompanied with a painful sensation of fullness on the affected fingers. Phalen’s test was positive, and electrophysiological tests were indicative of carpal tunnel syndrome due to median nerve compression. However, the ulnar nerve was not found entrapped through Guyon's canal. Likewise, the movements of middle and ring fingers were extremely restricted due to soft tissue hypertrophy and stiffness of his metacarpophalangeal and interphalangeal joints. The flexion of his two gigantic fingers was severely deteriorated due to malalignment of joints and angled phalanges. X-rays revealed excessive hand osteoarthritis with the presence of large bone spurs (osteophytes) and joint space narrowing between all phalanges of the third and fourth fingers. Therefore, a debulking reconstruction surgery of the overgrown fatty tissue and ray resection of the most enlarged fourth finger were performed along with carpal tunnel release.

**Figure 2:**
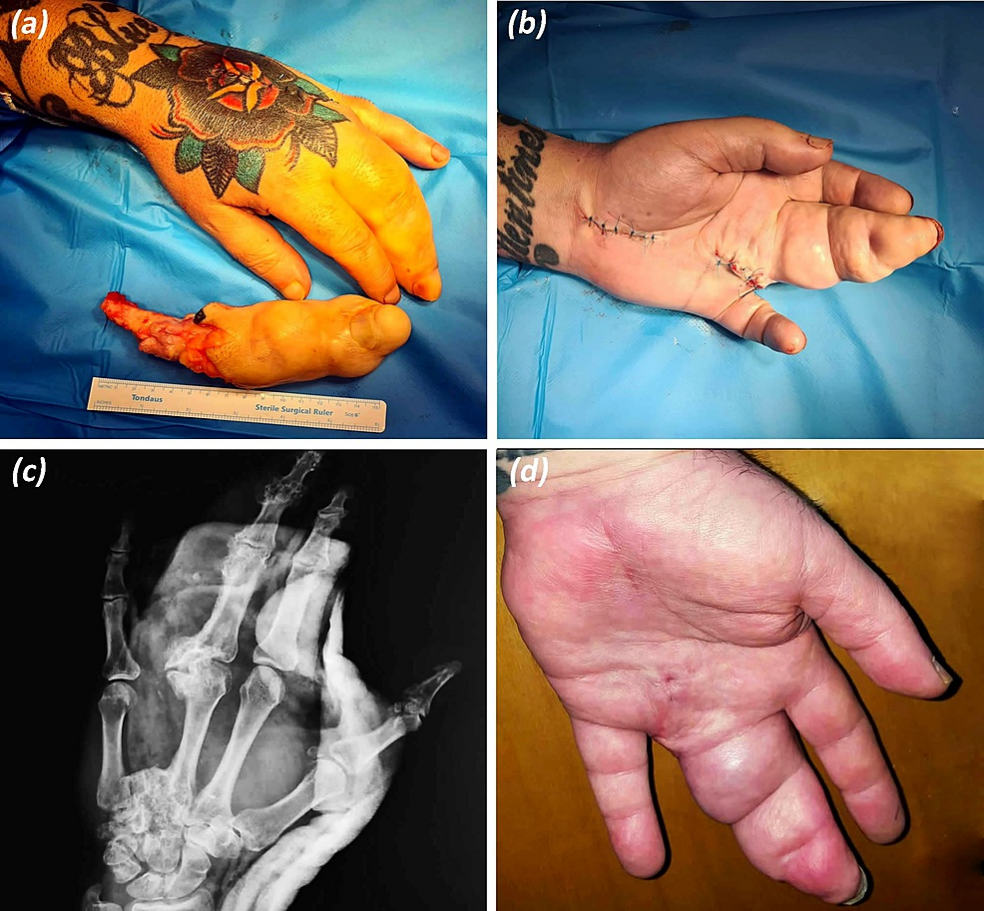
Postoperative images and at follow-up. (a) Ray resection at the proximal base of the fourth metacarpal. (b) Racket-shaped incision for the resection of the fourth ray and palmar incision for carpal tunnel release. (c) Postoperative X-ray with hand in splint. (d) Palmar view six months postoperatively, the patient reported good aesthetic and functional results.

Under axillary block anesthesia and application of a pneumatic tourniquet, a racket-shaped incision was made around the base of the fourth metacarpal. The extensor and flexor tendons (flexor digitorum superficialis and flexor digitorum profundus) as well as interosseous and lumbrical muscles of the fourth finger were detached and transected carefully to prevent tendon injuries of the remaining normal digits. Exposure of the digital neurovascular bundle of the third and fifth fingers was well visualized, mobilized, and preserved. Radial and ulnar digital nerves of the fourth finger were found enlarged and were easily recognized due to their increased thickness. Subsequently, nerve endings were carefully implanted within the surrounding soft tissue to avoid as much as possible the formation of painful neuromas. With a surgical oscillating saw, a transection at the base of the fourth metacarpal was performed. Subsequently, surgical debulking of the extensive soft tissue was implemented (Figure 2), and hemostasis of blood vessel’s leakage was achieved with an electrocautery. Eventually, median nerve decompression was achieved with a palmar incision to divide the transverse carpal ligament. The surgical procedure was performed by a senior consultant hand surgeon and a hand fellow orthopaedic surgeon. The postoperative plan consisted of temporary splinting and early supervised physiotherapy.

Postoperatively, the patient was complaining of phantom pain, which was well tolerated with a two-week prescription of acetaminophen and NSAIDs. Eventually, the phantom pain was resolved entirely eight weeks after surgery [5]. At the six-month follow-up, the patient reported a great recovery with excellent functional and aesthetic satisfaction (Figure 2). His grip strength and hand mobility improved with at least 30° of better flexion range for third metacarpophalangeal joint and so did abduction-adduction for his index, ring, and small fingers.

## Discussion

Until recently, pathogenesis of osseous and fibrofatty overgrowth in hand macrodactyly is not clearly identified and still no consensus exists on its treatment. Therefore, hand macrodactyly poses a significant surgical challenge. which often requires the expertise of experienced hand surgeons to manage effectively [6].

It has been reported that significant nerve enlargement is usually observed during surgical approach of affected digits with morphological and neurophysiological impairment of the median nerve that requires carpal tunnel release [7]. In the present case, the third and fourth rays were affected with simultaneous enlargement of the radial and ulnar digital nerve of the fourth finger. However, electrophysiological testing depicted entrapment of the median nerve but not of the ulnar nerve. Lipomatosis of peripheral nerves (fibrolipomatous proliferation within the nerve) accompanied with osseous enlargement and hypertrophic changes on osteochondral tissue leads not only to compressive neuropathy but also to disabling ankylosis of innervated joints [8].

Genetic studies have demonstrated that hand macrodactyly can be a clinical manifestation of three major overgrowth syndromes: the Proteus syndrome (mosaic mutations in the AKT1 gene), the PIK3CA-related overgrowth syndrome (mutations in the PIK3CA oncogene), and the PTEN hamartoma tumor syndrome (mutations in somatic PTEN tumor suppressor gene). According to Cui et al., somatic mutations in PIK3CA oncogene were observed within bone marrow stem cells from patients diagnosed with hand macrodactyly [9]. These specific mutations enhance activation of the PI3K/AKT/mTOR pathway and deregulate bone homeostasis, leading to hyperplastic bone formation [9]. Moreover, Cui et al. demonstrated that downregulation of distal-less homeobox 5 gene (DLX5) which induces Runx2-mediated osteogenesis and P13K-mediated bone overgrowth could be inhibited by the administration of BYL719 [9]. Subsequently, the administration of this novel therapeutic agent in early-stage disease could appropriately reverse progressive hand macrodactyly [9].

Progressive macrodactyly is an extremely challenging disease as no surgery is able to cure the underlying condition. Most patients, even if operated early in life, require multiple debulking procedures in accordance with the present case report. Concurring to the literature, only few published reports demonstrate potential strategies and surgical treatment options for progressive hand macrodactyly without a clear consensus on treatment guidelines, as shown in Table 1 [5,10]. Children with static hand macrodactyly can be appropriately treated with minimal surgical interventions such as stripping or resection of the local nerve, debulking, closing-wedge osteotomies, and phalangeal epiphysiodesis [10,11]. A very innovative surgical technique was proposed by Kobraei et al. to prevent fast skeletal overgrowth and avoid digits amputation in progressive hand macrodactyly [12]. According to the authors, a radical dissection of the diseased gross digital nerve in two cases with thumb (radial digital nerve) and ring finger (ulnar digital nerve) overgrowth was performed until healthy nerve stump was found [12]. The gaps were reconstructed with a processed nerve allograft between normal edges [12]. To author’s view, an early application of this novel surgical approach could yield functional and aesthetic digits with remarkable sensory outcomes and significant deceleration of the disease [12].

**Table 1:** Indicative surgical techniques in patients with hand macrodactyly. STD, soft tissue debulking; pts, patients; MCP, metacarpophalangeal

	Author/ year	Number of cases/age	Surgical interventions
1	Sumarwoto et al., 2021 [13]	1 case: macrodystrophia lipomatosa of middle finger; age: 14 years	STD of the entire tissue
2	Jacobs et al., 2020 [5]	1 case: right-hand thumb, index finger, and middle finger; age: 53 years	Amputation: removal of thumb and index rami, trapezoid, trapezium, and scaphoid. Resection of two exostoses from the capitate and palmar radius.
3	Wu et al., 2020 [2]	90 cases; age: 6 months to 25 years	STD: 12 pts; STD + digital nerve transection + anastomosis: 30 pts; STD + osteotomy: 17 pts; STD + digital nerve transection + osteotomy: 25 pts; STD + skin regrafting: 40 pts; amputation: 2 pts; carpal tunnel release: 3 pts; separation of syndactyly: 4 pts
4	Kobraei et al., 2019 [12]	2 cases; age: 4 years and 12 years; first case: left thumb and thenar macrodactyly; second case: ulnar-sided right ring finger macrodactyly	First case: segmental resection of radial digital nerve + radical STD of the radial side of thumb + nerve allograft; second case: ulnar digital nerve resection + STD + nerve allograft
5	Cavadas and Thione, 2018 [14]	2 cases; age: 2 years and 3years; first case: enlarged index and middle fingers, thumb, and ring finger; second case: left hand (enlarged index and middle fingers) and right hand (enlarged middle and ring fingers with syndactyly)	First case: resection of second ray and amputation of the middle finger (proximal metaphysis of middle phalanx) + ipsilateral second toe transfer; second case: left hand (resection of third ray + amputation of index finger + second toe transfer, epiphysiodesis for the ring finger and thumb) and right hand (resection of middle and ring finger syndactyly, amputation of third ray, fourth metacarpal, MCP joint, and base of the ring finger were preserved, reconstruction of ring finger with ipsilateral second toe)
6	Kakinoki et al., 2008 [15]	1 case: macrodactyly simplex congenita; age: 3 years	Fourth finger: epiphysial resection + osteosynthesis of distal interphalangeal joint longitudinal + transverse osteotomy of phalanges, soft tissue coverage with flaps
7	Akinci et al., 2004 [11]	5 cases; age: 12 to 32 years (mean: 17.5 years); first case: right thumb; second case: right thumb; third case: left index and middle fingers; fourth case: right ring finger; fifth case: left index and middle fingers	STD + shortening + narrowing of the distal phalanx and the middle phalanx + excision of the convex part of the distal interphalangeal joint
8	Krengel et al., 2000 [16]	4 cases; age: 6 months to 16 years	1 patient: amputation of the thumb 1 patient lost to follow-up (without operation); 1 patient lost to follow-up (refused surgery); 1 patient: amputation of the second, third, and fourth fingers

The largest case series study considering clinical characteristics and surgical management of 90 hand macrodactyly cases was conducted by Wu et al. [2]. According to their study, multiple digit involvement is up to 2.6 times more frequent than a single-digit disease, which is in line with our patient who had his middle and ring fingers enlarged. In the case series report by Wu et al., most of the affected digits (79.4%) involved were in the median nerve innervation surface [2]. However, in the present case report, the patient had a ring finger macrodactyly, which corresponds to the ulnar nerve area, with no signs of ulnar nerve compression. In addition, the study by Wu et al. included young patients aged between six months and five years. The vast majority of patients were treated with soft tissue reconstruction or minimal phalangeal osteotomies and only two out of 90 cases had an amputation [2]. Consequently, function-preserving surgeries are performed instead of amputation when hand macrodactyly is effectively treated during early-stage compared to advanced-stage disease.

Based on recent bibliography, Jacobs et al. presented the most advanced case of a 55-year-old female patient diagnosed with Proteus syndrome and macrodactyly of her right-hand thumb, middle, and index fingers [5]. The individualized surgical plan included amputation of the thumb and index rami and removal of trapezoid, trapezium, and scaphoid bones [5]. Consequently, the resulted wrist instability was treated with transosseous ligament reconstruction [5]. Good aesthetic and functional results were comparable to that in our patient who was treated with a lesser ray resection technique. To the best of our knowledge, the present case report depicts the surgical management of the oldest patient (60-year-old male) with progressive isolated macrodactyly among the published cases in recent literature. In addition, we strongly believe that efficient stabilization during early-stage disease would have prevented the development of severe chronic osteoarthritis in the metacarpophalangeal, proximal interphalangeal, and distal interphalangeal joints of the third and fourth fingers, which add more disability to a macrodactyly hand.

## Conclusions

Hand macrodactyly is usually visible at birth and patients experience overgrowth symptoms during early childhood. Consequently, an early and effective surgical management is strongly recommended to prevent chronic progression and development of severe secondary degenerative bone changes in macrodactyly fingers, such as ankyloses, narrowing of joints, and formation of osteophytes. Patients suffering from hand macrodactyly can significantly benefit from early surgical stabilization of the condition instead of late and more aggressive interventions such as amputation.

In the present case, surgical interventions at an early stage proved ineffective, and the patient developed severe and disabling hand deformities due to the progressive subtype of hand macrodactyly. Most of the macrodactyly cases seem to stabilize at skeletal maturity, and it is unusual to see this degree of progressive bony overgrowth. Nevertheless, a personalized surgical technique including ray resection and debulking reconstruction surgery was proposed for this neglected case with great aesthetic and functional outcomes.
